# A rare clinical presentation of metronidazole-induced dysarthria: A Case report with literature review

**DOI:** 10.1016/j.radcr.2025.02.042

**Published:** 2025-03-08

**Authors:** Arpan Mitra, Niraj Kumar Srivastava, Ankur Vivek, Nayana Bhuyan, Akansha Jain, Subhraneel Paul, Vijaya Nath Mishra, Abhishek Pathak

**Affiliations:** a Department of Neurology, Institute of Medical Sciences (IMS), Banaras Hindu University (BHU), Varanasi 221005 Uttar Pradesh, India; b Department of Radiology, Tripura Medical College (TMC), Agartala 799130 Tripura, India

**Keywords:** Dysarthria, Metronidazole, Liver abscess, Central nervous system, Cerebellum, Antibiotic, Encephalopathy

## Abstract

We described a case of a rare, but notable adverse effect of metronidazole therapy, where a 55-year-old chronic alcoholic patient developed dysarthria following a four-week course of the drug for the treatment of pyogenic liver abscesses. Dysarthria, characterized by slurred or unclear speech, is an uncommon complication of metronidazole, which is generally well-tolerated but has the potential to cause neurotoxic effects in some individuals, especially with prolonged use or high cumulative doses. In this case, the Magnetic Resonance Imaging (MRI) brain revealed T2/FLAIR hyperintensities involving the bilateral dentate nuclei, a key finding associated with metronidazole-induced encephalopathy. The exclusion of other possible causes for dysarthria led to the conclusion that the symptoms were likely induced by metronidazole. This highlights the importance of considering drug-induced neurotoxicity in patients presenting with new neurological symptoms, particularly when there is a history of prolonged antibiotic therapy. The patient's improvement after discontinuing metronidazole and switching to alternative treatment further supports this diagnosis. This case underscores the necessity of closely monitoring patients on metronidazole, especially those receiving prolonged treatment, for any emerging neurological signs. Typical MRI findings play a crucial role in the vigilance for the diagnosis and treatment of such clinical situations. Timely recognition and intervention can help prevent permanent damage and facilitate recovery.

## Introduction

Metronidazole is a commonly prescribed antibiotic for treating infections caused by parasites and anaerobes. This medication can cause serious adverse reactions and involvement of both the central nervous system and the peripheral nervous system, in addition to common side effects including nausea and abdominal pain. Metronidazole-induced encephalopathy (MIE) is the term used to describe such significant unfavorable effects on the central nervous system. The symptoms of MIE, a unique syndrome of cerebellar dysfunction, include nystagmus, dysarthria, ataxia, and dysmetria [[1], [2], [3], [4], [5]]. The dentate nuclei are two deep structures in the cerebellum that are essential for motor control, and imaging examinations frequently show abnormalities in these areas. When combined with clinical symptoms, neuroimaging aids in the diagnosis of MIE. It is an uncommon but treatable side effect linked to long-term metronidazole treatment. The longer the therapy is administered and the higher the dose, the higher the chance of MIE. With symptoms ranging from headaches and altered mental status to convulsions and focal neurological deficits, the illness primarily affects the cerebellum. Although these side effects usually subside when metronidazole is discontinued, the recovery rate differs in each patient [[3], [4], [5], [6]]. A patient in one MIE case report was admitted with a brain abscess and needed prolonged antibiotic treatment. The significance of keeping an eye out for side effects, particularly neurotoxicity, is shown by the patient's developing neurological symptoms during extended metronidazole medication. Preventing irreversible harm and enhancing patient outcomes need early detection and timely treatment, which includes stopping the offending drug and switching to substitute antibiotics [[5], [6], [7]]. In a different case report, a patient received metronidazole treatment after being diagnosed with a liver abscess. After a month of therapy, he started to experience neurological symptoms, most likely from MIE, which went away after the medication was stopped. A thorough analysis and description are carried out in the context of the MIE in one systematic review. This study also emphasizes the potential for recovery upon stopping the medication, assuming no serious comorbidities are worsening the condition, and stresses the significance of keeping an eye out for symptoms of polyneuropathy in patients taking metronidazole, particularly if they had pre-existing liver illness [[4], [5], [6], [7], [8], [9]].

In light of all the above descriptions of MIE and its significance in the context of the patient's treatment, we are also going to add this metronidazole-induced cerebellar toxicity-based case report. Here, we presented a case of a chronic alcoholic patient with multiple liver abscesses (pyogenic), who received metronidazole and developed slurring of speech, which is a unique and rare side effect.

## Case report

A male patient, aged 55, was hospitalized with a complaint of fever (low grade, intermittent type) for the last 15 days. He also complained of pain in the right upper abdomen which is mild to moderate in intensity. There was no history of diarrhea or vomiting, burning micturition, chest pain, cough, or dyspnea. Fever was not associated with joint pain, skin rashes, or bleeding manifestations. He had no prior comorbidity, but he was a habitual consumer of alcohol (70-80 grams/day) for the last 10 years. On evaluation, he was diagnosed with multiple liver abscesses (pyogenic) and treatment was started with a combination of metronidazole (2.4 gm/day in divided doses), meropenem, and other supportive medications. With these treatments, there was an improvement in his symptoms and also relevance for the discharge of the patient from the hospital. He was followed up in the outpatient department and a serial ultrasound abdomen showed a reduction in the size of the abscesses. But 4 weeks after starting medication, he presented to the neurology department with a 3-day history of acute onset, progressive slurring of speech. He did not complain of gait changes, swaying of hand while approaching an object, tremulousness, or difficulty in doing day-to-day activity neither did he have confusion, altered sensorium, motor weakness, paraesthesia, or burning sensation.

The initial clinical and physical examinations were performed at the time of admission. It was found that he is febrile (100.5 F), with a pulse rate of 102 bpm and a respiratory rate of 18 breaths/min. Blood pressure was 112/68 mm Hg. Oxygen saturation (SPO_2_) 96% in room air. Other general physical examinations were absent. Per-abdominal examination showed tenderness in the right hypochondrium and hepatomegaly. The central nervous system, cardiovascular system, and chest examination were within normal limits. However, on follow-up, when the patient presented with dysarthria, his speech was found to be scanning and explosive type. The patient's sole complaint was slurred speech, but upon examination, minor dysmetria and impaired tandem walking were found to be subtle additional cerebellar symptoms.

**Fig. 1:**
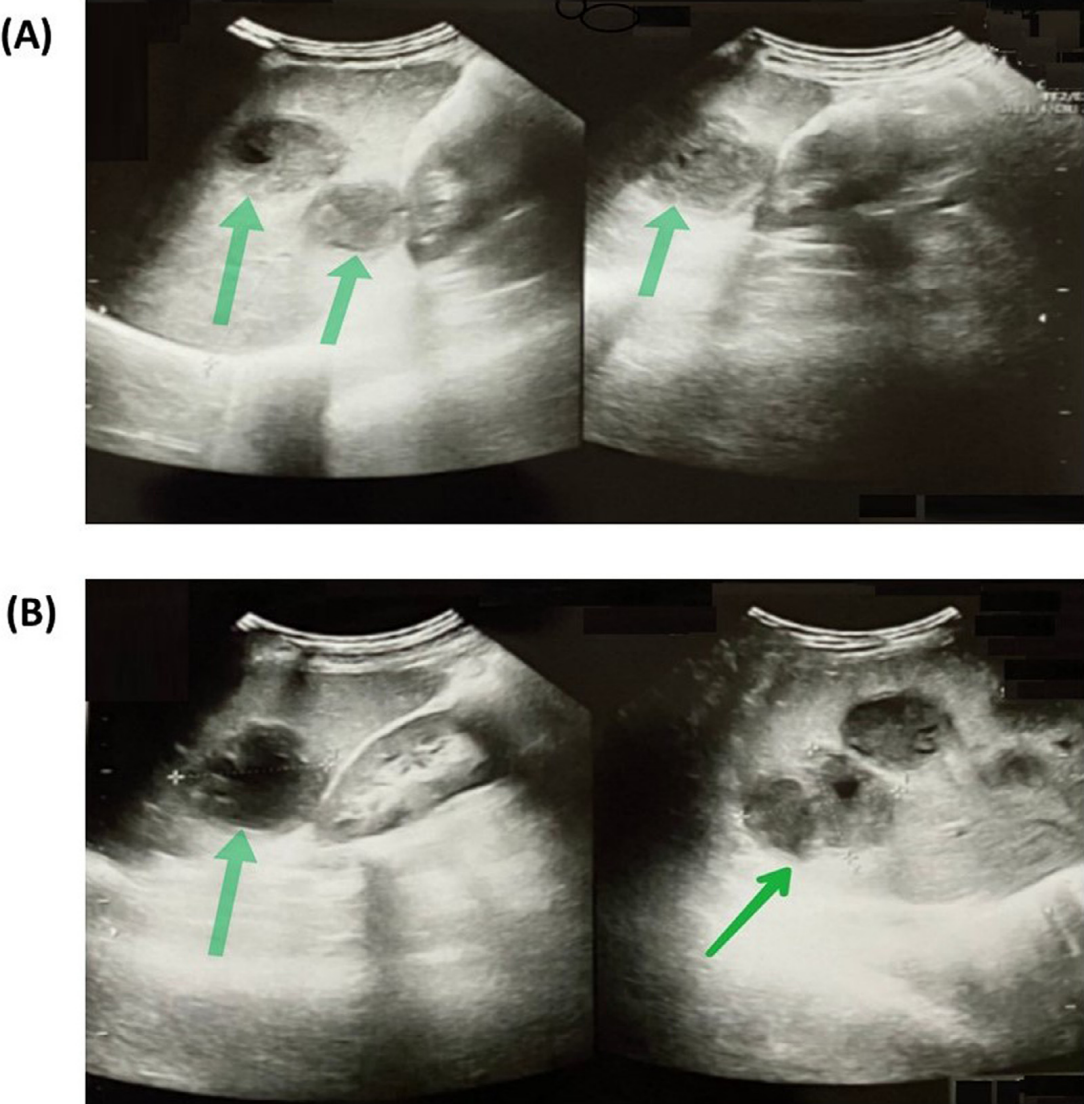
Multiple liver abscesses (pyogenic) are represented by USG abdomen (ANB) (Green colour arrows indicated the abscesses in images [A] and [B]).

**Fig. 2:**
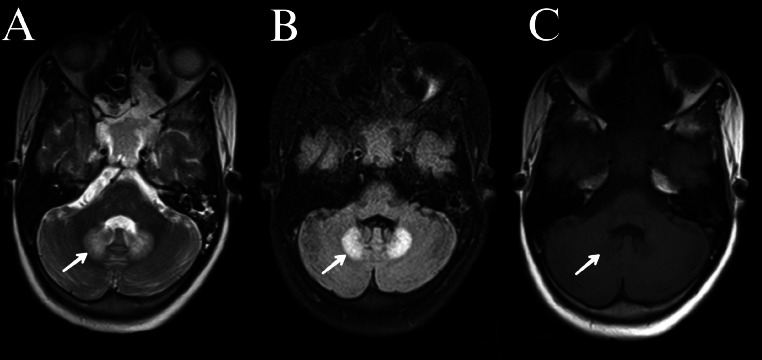
MRI images of the brain at the level of middle cerebellar peduncle shows T2 (A) and FLAIR (B) hyperintensity (indication with white arrow) of bilateral dentate nuclei with T1 (C) hypointensity (indication with white arrow).

Numerous blood-based investigations, aspirated pus analysis, urine, and other laboratory-based examinations were performed (Table 1-5 are in the supplementary files). Ultrasound imaging of the liver is represented in Fig. 1. MRI brain is represented by Fig. 2. The outcomes of the nerve conduction study (Fig. 3 is in the supplementary file) suggest a normal finding.

Diagnosis of multiple pyogenic liver abscesses is established by the findings of the ultrasound-based imaging of the liver. The MRI brain showed T2 and FLAIR hyperintensity of bilateral dentate nuclei of the cerebellum with T1 hypointensity, without diffusion restriction or contrast enhancement, which is indicative of metronidazole-induced cerebellar toxicity.

The patient was treated for multiple liver abscesses with a prolonged, high-dose regimen of metronidazole. Subsequently, he developed cerebellar dysarthria along with other subtle cerebellar signs. MRI findings played a crucial role in confirming the diagnosis. Recognizing the adverse effect, metronidazole was promptly discontinued. Alternative antibiotic therapy was initiated with amikacin, guided by the pus culture and sensitivity report. Following the discontinuation of metronidazole, the patient's neurological symptoms improved significantly on follow-up, which is characteristic of the reversible nature of this drug-induced toxicity.

## Discussion

We presented a case of a patient with multiple pyogenic liver abscesses who developed dysarthria after 4 weeks of metronidazole treatment. This is an important observation, as dysarthria, though a rare side effect, has been associated with prolonged or high-dose metronidazole therapy [1,2,5]. Metronidazole is an extensively utilized 5-nitroimidazole compound, predominantly efficient against anaerobic bacteria and protozoal infections [1]. Its mechanism entails the reduction of its nitro group in anaerobic cells, which produces reactive nitro radicals that destroy DNA and other vital biological functions, ultimately resulting in cell death. Because anaerobic organisms have unique metabolic pathways that allow for this kind of mechanistic action, metronidazole is a targeted and generally safe therapeutic approach for pathological conditions like trichomoniasis, amoebiasis, giardiasis, bacterial vaginosis, and some anaerobic bacterial infections [[1], [2], [3], [4], [5], [6]]. The most frequent side effects of this medication include nausea, vomiting, metallic taste, vertigo, weakness, and peripheral neuropathy. However, ataxia, dysarthria, and disturbed mental status are far less common symptoms. According to the currently available literature, the symptoms of metronidazole-induced cerebellar dysarthria, which we also documented, are quite rare and exceptional [1,2,[4], [5], [6]]. An MRI brain scan of this patient revealed T2/FLAIR hyperintensities in both dentate nuclei (Fig. 2). This MRI feature is both distinctive and significant in the context of the diagnosis [6,[10], [11], [12], [13], [14], [15]]. Several other conditions can mimic this MRI finding and clinical presentation, and it's important to consider these in the differential diagnosis. These are demyelination, Wernicke's encephalopathy, Marchiafava-Bignami disease, space-occupying lesions, vascular events, postictal state, CNS TB, syphilis, HIV infection, other antibiotic-associated encephalopathies (penicillin, cephalosporins, sulphonamides, fluoroquinolones, and macrolides), primary CNS lymphoma and progressive multifocal leukoencephalopathy (Table 5; Supplementary Files) [5,6,9,16]. Moreover, inborn errors of metabolism, lysosomal storage diseases, and mitochondrial diseases can present with similar clinical and radiological features but they can be differentiated by their typically childhood onset and slower progression [16]. MIE, on the other hand, is a clinical and radiological diagnosis of exclusion. When other causes are ruled out, a temporal association of symptom development and MRI changes with metronidazole use, along with their reversal upon cessation of the drug, can provide sufficient evidence for diagnosing this condition [[1], [2], [3], [4], [5],^6^]. Metronidazole is also known to cause peripheral neuropathy mainly small fibre neuropathy. The findings of dysesthesia, paraesthesia, burning sensation, diminished to absent deep tendon reflexes, and impaired tandem walking suggest metronidazole-induced neuropathy. In this case, the normal findings of the nerve conduction study (Fig. 3) ruled out PNS involvement, further supporting the diagnosis of isolated CNS toxicity. All these findings are also supported by the previously reported case of MIE [9].

Based on research-based studies and case reports, it is stated that metronidazole can cause neurotoxicity, especially at higher cumulative doses or prolonged treatment durations. The risk increases significantly beyond 42 grams of cumulative dose or treatment durations exceeding 4 weeks [5,6,17,18]. In our reported case, the overall dose of metronidazole consumed by the patient was 67.2 grams and the treatment duration was 4 weeks. This again correctly overlapped with the above-mentioned facts of existing literature. The pathogenesis of MIE remains under investigation, with several hypotheses proposed. One hypothesis suggests that methylation of intracellular proteins and lipids by metronidazole could disrupt essential metabolic processes, akin to chronic methyl bromide intoxication. Another theory posits that metronidazole may interfere with energy metabolism, particularly affecting vulnerable areas like the cerebellum, brainstem, and periventricular regions [5,19,20]. Additionally, inhibition of protein synthesis and vasospasm induced by metronidazole metabolites binding to neural cell DNA or RNA have been suggested as potential mechanisms. Whether MIE is dose-dependent or idiosyncratic remains unclear and is a subject of research [5,19,20].

MIE is a serious, but uncommon condition associated with prolonged use of metronidazole. It typically manifests with cerebellar dysfunction and MRI findings often show signal changes in the dentate nuclei. MRI is the preferred imaging modality over CT scans due to its superior sensitivity and safety in diagnosis [21] of MIE as performed in the previous studies [[1], [2], [3], [4], [5], [6], [7],^16^]. Diagnosis is primarily performed and established by MRI-based imaging and ruling out other potential causes, and discontinuation of metronidazole usually leads to reversal of symptoms. While metronidazole is generally considered safe, awareness among clinicians about these rare adverse effects is crucial for early recognition and management. By recognizing the CNS toxicity of metronidazole, our presented case report will be adding to the previously reported cases in the literature.

## Limitations

The study is based on a single clinical case, limiting the generalizability of findings to a broader population. No other biochemical or neuroimaging biomarkers were explored beyond MRI to identify this specific abnormality.

## Future scope

Further studies are needed to better understand the prevalence and clinical spectrum of metronidazole-induced dysarthria. Future research should focus on the exact mechanisms, including oxidative stress, mitochondrial dysfunction, and neurotransmitter alterations, that contribute to metronidazole neurotoxicity. Prospective studies incorporating multimodal neuroimaging (MR spectroscopy, functional MRI, DTI [diffusion tensor imaging]) can provide deeper insights into structural and functional brain changes. Studies assessing cumulative metronidazole dosage thresholds leading to dysarthria can aid in safer prescribing practices. The development of standardized diagnostic criteria and early intervention strategies is needed for metronidazole-induced neurological toxicity.

## Patient consent

We confirm that we have obtained written, informed consent from the patient for the publication of this case report. The patient has been thoroughly informed about the details that will be published and understands the implications of the publication. The written consent is stored securely and is available for review by the editorial team upon request.

## Appendix Supplementary materials

Image, application 1

Video 1: Patient showing dysarthria (particularly the scanning type, which is characterized by slow and irregular speech with variable syllable stress, often suggests cerebellar involvement). Image, video 2
