# Supplementary material for: A rare clinical presentation of metronidazole-induced dysarthria: A Case report with literature review
Source: Radiol Case Rep. 2025 Mar 8;20(5):2355–9. doi: 10.1016/j.radcr.2025.02.042 (PMC11930524; doi:10.1016/j.radcr.2025.02.042)
Supplement: Supplementary file 1 [file mmc1.docx]

**Table-1.** **Blood investigation of the patient is represented**

| **S.No.** | **Components** | **Quantitative value** |
| --- | --- | --- |
| 1. | Hb% (g/dL) | 11.2 |
| 2. | TLC (per cumm) | 20900 |
| 3. | DLC (N %/L%) | 89/3.5 |
| 4. | MCV (fL) | 112 |
| 6. | Platelet (per cumm) | 225000 |
| 7. | Creatinine (mg/dL) | 1.6 |
| 8. | Urea (mg/dL) | 95 |
| 9. | Na+ (mmol/L) | 125 |
| 10. | K+ (mmol/L) | 5.9 |
| 11. | SGOT (IU/L) | 134 |
| 12. | SGPT (IU/L) | 205 |
| 13. | T.BIL (mg/dL) | 2.9 |
| 14. | D.BIL/I.BIL (mg/dL) | 2.3/0.6 |
| 15. | Total Protein (g/dL) | 5.9 |
| 16. | S. ALB (g/dL) | 3.5 |
| 17. | ALP (IU/L) | 311 |
| 18. | RBS (mg/dL), HbA1c | 140, 5.6 |
| 19. | PO4 (mg/dL) | 3.3 |
| 24. | Ca2+ (mg/dL) | 9.7 |
| 25. | ESR (mm/hour) | 120 |
| 26. | qCRP (mg/dL) | 0.63 |
| 27. | PT/INR | 20.6/1.58 |
| 28. | Blood culture & sensitivity | Sterile |
| Thyroid profile | | |
| 29. | Serum T3 (ng/ml)  Serum T4 (ug/dl)  Serum TSH (uIU/ml) | 0.87  6.6  1.02 |

**Table-2. Representation of the aspirated pus analysis**

| **Analysis of the pus aspirated from liver abscess** | | | |
| --- | --- | --- | --- |
|  | **Physical Examination** | | |
| **S.No.** | **Parameters** | **Qualitative and quantitative outcomes** | |
| 1. | Volume | 4.50 ml | |
| 2. | Colour | Brownish | |
| 3. | Turbidity | Positive | |
| 4. | Coagulum | Nil | |
| 5. | Blood | Nil | |
| 6. | Deposit | Slight | |
|  | Chemical Examination | | |
| 1. | Glucose | | 186.00 mg/dl |
| 2 | Chloride | | 81.10 mmol/L |
| 3. | Total Protein | | 2.39 g/dL |
|  | Cytological Examination | | |
|  | Cell count | | 410.00/ mm3 |
|  | Neutrophils | | 10.00% |
|  | Lymphocytes | | 90.00% |
| pus culture and sensitivity | | | Pseudomonas aeruginosa- heavy growth sensitive to amikacin, cefepime, meropenem. |

**Table-3.** **Urine examination is represented**

| **S.No.** | **Components** | **Result** |
| --- | --- | --- |
| 1. | Protein | 75 mg/dl |
| 2. | Sugar | Nil |
| 3. | Pus cells | 15-20/HPF |
| 4. | RBC | 50-60/HPF |
| 5. | Ketone | 50 mg/dl |
| 6. | C/S | Sterile |

**Table-4.** **Other investigations are represented**

| **S.No.** | **Investigation** | **Result** |
| --- | --- | --- |
| 1. | CXR AP view | Normal |
| 2. | USG abdomen | Hepatomegaly with multiple partly liquefied abscesses in the right lobe of the liver, minimal ascites |
| 3. | ECG | Sinus tachycardia |
| 4. | 2 D ECHO | Normal |
| 6. | Viral markers (HIV-1 & 2, HBsAg, anti-HCV) | Non-reactive |
| 7. | Fundoscopy | Normal |
| 9. | Vasculitis panel (ANA, ANCA) | Negative |
| 11. | Serum ACE level (U/L) | 29 |
| 12. | S. VDRL | Non-reactive |
| 13. | Malaria para check | Negative |
| 14. | IgM dengue, NS1 antigen | Negative |
| 15. | IgM typhoid | Negative |
| 16. | IgM scrub | Negative |
| 17. | IgM Leptospira | Negative |

**Table-5. Summary of the Explanatory Differential Diagnosis of MRI**

| **S.No.** | **T2/ FLAIR images [hyperintensities of dentate nucleus]** | **Disease /pathological conditions** |
| --- | --- | --- |
| **1.** |  | **Metronidazole-induced cerebellar toxicity** |
| **2.** |  | **Demyelination** |
| **3.** |  | **Wernicke’s encephalopathy** |
| **4.** |  | **Marchiafava-Bignami disease** |
| **5.** |  | **Space-occupying lesions** |
| **6.** |  | **Vascular events** |
| **7.** |  | **Postictal state** |
| **8.** |  | **CNS TB** |
| **9.** |  | **Syphilis** |
| **10.** |  | **HIV infection** |
| **11.** |  | **Other antibiotic-associated encephalopathies (penicillin, cephalosporins, sulphonamides, fluoroquinolones, and macrolides)** |
| **12.** |  | **Primary CNS lymphoma** |
| **13.** |  | **Progressive multifocal leukoencephalopathy** |
| **14.** |  | **Maple syrup urine disease (MSUD)** |
| **15.** |  | **Glutaric aciduria type 1 (GA-1)** |
| **16.** |  | **Canavan disease** |


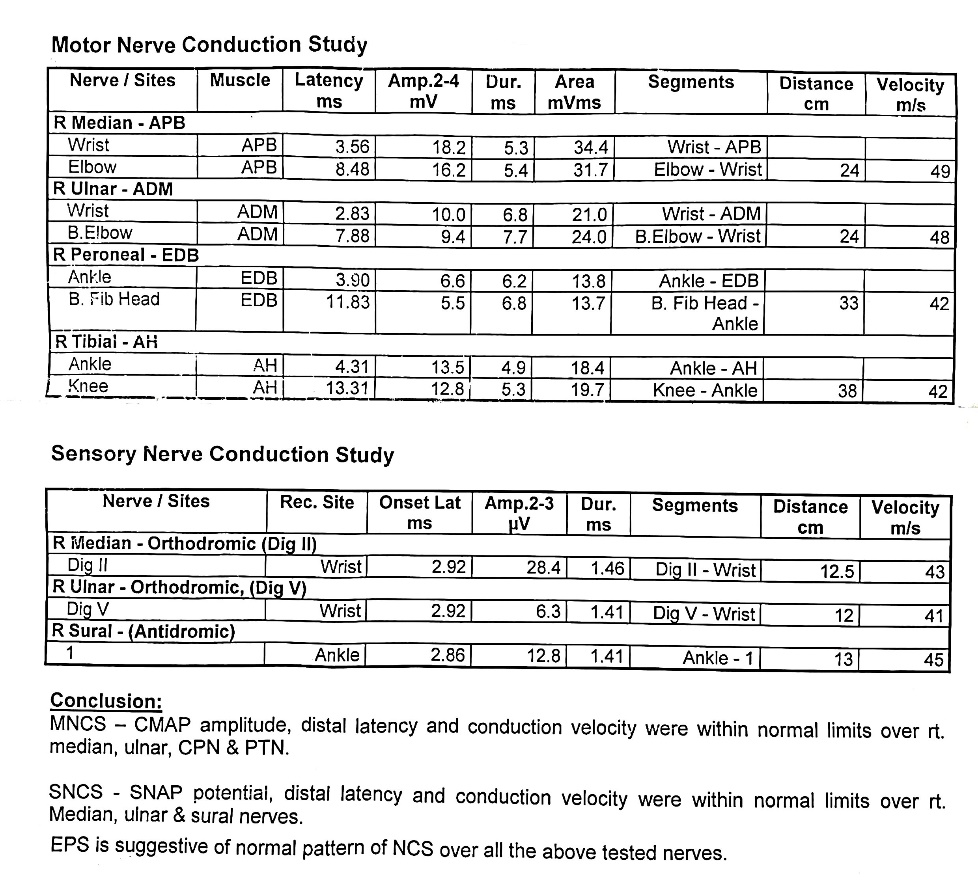


**Figure-3.** Nerve conduction study of the upper limb and lower limb are showing normal pattern in all tested nerve [Supplementary Files].
